# Identification of a ERCC5 c.2333T>C (L778P) Variant in Two Tunisian Siblings With Mild Xeroderma Pigmentosum Phenotype

**DOI:** 10.3389/fgene.2019.00111

**Published:** 2019-02-14

**Authors:** Asma Chikhaoui, Sahar Elouej, Imen Nabouli, Meriem Jones, Arnaud Lagarde, Meriem Ben Rekaya, Olfa Messaoud, Yosr Hamdi, Mohamed Zghal, Valerie Delague, Nicolas Levy, Annachiara De Sandre-Giovannoli, Sonia Abdelhak, Houda Yacoub-Youssef

**Affiliations:** ^1^Laboratoire de Génomique Biomédicale et Oncogénétique, Institut Pasteur de Tunis, Université de Tunis El Manar, Tunis, Tunisia; ^2^Aix Marseille Univ, Inserm, MMG, U 1251, Marseille, France; ^3^Service de Dermatologie, Hôpital Charles Nicolle, Tunis, Tunisia; ^4^Département de Génétique Médicale, AP-HM, Hôpital la Timone, Marseille, France

**Keywords:** Xeroderma pigmentosum-G, NER defects, aging, cancer, XP/cockayne syndrome

## Abstract

Xeroderma pigmentosum (XP) is a rare autosomal recessive disorder due to a defect in the nucleotide excision repair (NER) DNA repair pathway, characterized by severe sunburn development of freckles, premature skin aging, and susceptibility to develop cancers at an average age of eight. XP is an example of accelerated photo-aging. It is a genetically and clinically heterogeneous disease. Eight complementation groups have been described worldwide. In Tunisia, five groups have been already identified. In this work, we investigated the genetic etiology in a family with an atypically mild XP phenotype. Two Tunisian siblings born from first-degree consanguineous parents underwent clinical examination in the dermatology department of the Charles Nicolle Hospital on the basis of acute sunburn reaction and mild neurological disorders. Blood samples were collected from two affected siblings after written informed consent. As all mutations reported in Tunisia have been excluded using Sanger sequencing, we carried out mutational analysis through a targeted panel of gene sequencing using the Agilent HaloPlex target enrichment system. Our clinical study shows, in both patients, the presence of achromic macula in sun exposed area with dermatological feature suggestive of Xeroderma pigmentosum disease. No developmental and neurological disorders were observed except mild intellectual disability. Genetic investigation shows that both patients were carriers of an homozygous T to C transition at the nucleotide position c.2333, causing the leucine to proline amino acid change at the position 778 (p.Leu778Pro) of the *ERCC5* gene, and resulting in an XP-G phenotype. The same variation was previously reported at the heterozygous state in a patient cell line in Europe, for which no clinical data were available and was suggested to confer an XP/CS phenotype based on functional tests. This study contributes to further characterization of the mutation spectrum of XP in consanguineous Tunisian families and is potentially helpful for early diagnosis. It also indicates that the genotype-phenotype correlation is not always coherent for patients with mild clinical features. These data therefore suggest that targeted NGS is a highly informative diagnostic strategy, which can be used for XP molecular etiology determination.

## Introduction

Xeroderma pigmentosum (XP) is an autosomal recessive genetic disorder that affects the Nucleotide Excision Repair (NER) DNA repair pathway with an incidence of 1/250.000 in Europe (Kleijer et al., 2008). However, it is more frequent in North Africa and especially in Tunisia (1:10.000) due to high rate of consanguinity (Zghal et al., 2005). Patients are characterized by UV-induced skin depigmentation, sunburn, skin cancers, premature skin photo-aging, and, in some patients, neurological degeneration. Genetically, it is assigned to eight complementation groups (XP-A-G, and XP-V). Deep clinical and molecular analyses showed that the correlation between phenotype and genotype is generally difficult in these diseases and revealed heterogeneity between and within complementation groups. This evidence makes the genetic diagnosis, using conventional sequencing techniques, costly, and time consuming despite the dominance of certain forms of XP in some geographical areas facilitates the orientation of the genetic tests, as we previously described (Jerbi et al., 2016).

Since, we started studying XP we reported five complementation groups (XP-A, C, D, E, and V) in Tunisia. The mutations with founder effect responsible for the XP-C (Ben Rekaya et al., 2009) and XP-A phenotypes (Messaoud et al., 2010) have been also found in other North African countries (Soufir et al., 2010) such as Morocco (Senhaji et al., 2013; Kindil et al., 2017), Egypt (Amr et al., 2014), Algeria (Bensenouci et al., 2016), and Libya (unpublished data). The XP-D and XP-E forms have been recently identified thanks to whole exome sequencing (WES) technology (Ben Rekaya et al., 2018). Patients belonging to these last two groups had mild dermatological manifestations, absence of neurological anomalies, and late onset of skin tumors. Moreover, these forms have been incorrectly assigned as XP-V, since the three phenotypes share the same clinical characteristics (Ben Rekaya et al., 2014).

In this work, we describe a family native of a region in Tunisia, known for hosting mainly the XP-A form (31 patients XP-A and three patients XP-C). Sanger sequencing failed to detect mutations associated with these phenotypes. Conversely, using targeted gene sequencing we identified a novel homozygous mutation related to one of the rarest forms of XP in the world: the XP-G complementation group.

Excision Repair Cross-Complementation Group 5 (*ERCC5, XPG*, OMIM 278780) gene is mapped to chromosome 13q32.3-q33 and contains 15 exons (Emmert et al., 2001). The first case assigned to the XP-G group was reported in 1979 by Keijzer et al. (1979). Mutations in this gene cause Xeroderma pigmentosum complementation group-G (XP-G), characterized by hypersensitivity to UV light and increased susceptibility to skin cancer development, with an average age of tumor onset at around the age of 20 (Natale and Raquer, 2017). The XP-G form has not been previously reported in North Africa.

The XPG protein has DNA endonuclease activity without preference for damaged DNA and is responsible for the 3′ incision made during NER. Patients belonging to the XP-G complementation group clinically exhibit heterogeneous symptoms, which range from mild to very severe phenotype. To date, the *ERCC5* mutations reported result in either XP-G or in a combined XP/Cockayne syndrome (XP/CS) phenotype (Ichihashi et al., 1985; Nouspikel and Clarkson, 1994; Nouspikel et al., 1997; Okinaka et al., 1997; Zafeiriou et al., 2001; Emmert et al., 2002; Lalle et al., 2002; Moriwaki et al., 2012; Soltys et al., 2013; Fassihi et al., 2016; Zhang et al., 2017; Zhou et al., 2017). The combined XP/CS is characterized by severe growth defects, microcephaly, cognitive disability, premature aging, dwarfism, and skin atrophy in addition to the XP disease (Ito et al., 2007).

In this work, we report for the first time the XP-G form in the Tunisian population, which is characterized by a mild XP phenotype associated with premature skin aging.

## Case Presentation

### Clinical Report

Patients (XP174-1 and XP174-2) are siblings originating from Kasserine (Central-West), in Tunisia. They were enrolled by the Dermatology Department of Charles Nicolle Hospital because of repeated sunburns, thanks to the regular campaigns conducted by the patients support group “Helping Xeroderma pigmentosum Children” to raise awareness on cancer risks related to skin photosensitivity.

**FIGURE 1:**
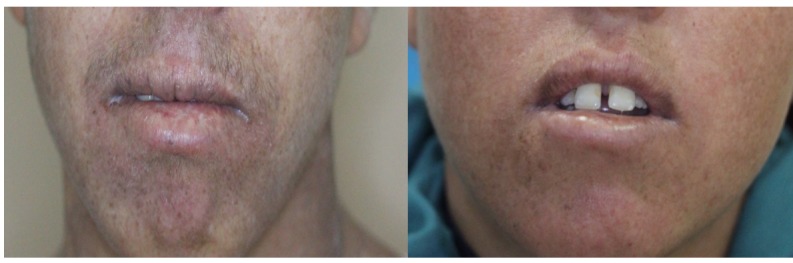
Clinical features of XP174-1 and XP 174-2. Hyperpigmented macules in exposed area and marked cheilitis in both patients.

The proband, a 21-year-old girl (XP174-1), and her 17-year-old brother (XP174-2) were born from first-degree consanguineous parents. Despite living in the Central-Western region of Tunisia (Kasserine), where they were often exposed to sunlight, they showed normal growth development, no dwarfism, nor sensorineural deafness or microcephaly that could rather suggest to be affected by the Cockayne syndrome. At the last consultation the patients were in good general health. Both siblings had achromic macules in sun-exposed areas (Table 1 and Figure 1). They also presented photosensitivity with acute sunburns. Both patients had no evidence of premalignant skin lesions or a history of skin cancer, but slight neurological deficiency. Indeed, the girl dropped out of school at the age of 12, while her brother was still in school but had learning difficulties that may reflect these mild neurological abnormalities. Their overall clinical presentation suggested a mild XP phenotype with slight neurological deficiency rather than a severe combined XP/Cockayne syndrome profile.

**Table 1:** Clinical features of XP-G patients.

Patients	XP174-1	XP174-2
Gender	F	M
Geographic origin	Kasserine	
Age	22	17
Sun protection level	-	-
Intellectual disability	+/-	+/-
Psychomotor retardation	+	-
Photophobia	+/-	+/-
Acute sunburn reaction	+	+
Hyperpigmented macules in exposed area	+	+
Hypopigmented macules in non-exposed area	+	+
Achromic macules in exposed area	+	+
Xerosis	+	+
Cheilitis	+	+
Skin cancer	-	-
Failure to thrive	-	-
Microcephaly	-	-


Only the father of the affected siblings was present during the consultation, he did not present any abnormality and stated that the mother was also in good health. However, during the genetic inquiry he indicates that other family members might have similar clinical phenotype as his two children. Unfortunately, these individuals were out of reach.

## Materials and Methods

### DNA Samples

After obtaining written informed consent from legal tutor of the patient, clinical and genealogical information were collected as well as peripheral blood samples from the two patients and from their father (XP174-3), who was the only available parent. Genomic DNA was extracted from peripheral blood samples using salting-out method and purified by QIAamp DNA Micro (Qiagen).

This study was conducted according to the principles of the declaration of Helsinki and has obtained the ethics approval (IPT/LR05/ProjectPCI/22/2012/v2) from the institutional review board of Pasteur Institute of Tunisia, Registration number (IRB00005445 and FWA00010074). All subjects gave written informed consent for the publication of clinical and laboratory data.

### HaloPlex Library Preparation and Sequencing

Custom design of the genes’ panel was performed using SureDesign (Agilent Technologies Inc.); probes were generated to cover the exons and 15 bp of the surrounding intronic sequences of a total of 87 candidate genes known to be involved in DNA repair disorders (Supplementary Table S2). Library preparation for NGS was performed using the Agilent’s HaloPlex^HS^ (high sensitivity) workflow as a target enrichment method. Amplicon libraries were prepared from patients’ genomic DNA using the HaloPlex^HS^ PCR target enrichment system dedicated to Ion Torrent PGM according to the manufacturer’s recommendations. Massively parallel sequencing was performed on an Ion Torrent PGM (Thermo Fisher Scientific).

### HaloPlex Bioinformatics Analysis

Raw data generated by the PGM sequencer was processed by Torrent Suite Software v.4 and aligned using TMAPv.3. Sequence variants were identified using the Variant Caller tool from the Ion Torrent package using default “germline low stringency” parameters (min_cov_each_strand: 0, min_variant_score: 10, min_allele_freq: 0.1, snp_min_coverage: 6 snp and indel; strand_bias: 0.98 snp and 0.85 indel) analyzed using the in-house VarAft software version 2.5, which is available online^1^. We prioritized rare functional variants (missense, nonsense, splice site variants, and indels) and excluded variants with a Minor Allele Frequency (MAF)>0.01 in dbSNP137 and 138, in the Exome Variant Server^2^, 1000 Genomes Project^3^, or Exome Aggregation Consortium database (ExAC), Cambridge, MA, United States^4^. A number of online tools were used to predict the functional impact and pathogenicity of the variants such as MutationTaster^5^, PolyPhen^6^, SIFT^7^, and I-mutant^8^.

### Mutation Confirmation by Sanger Sequencing

Polymerase chain reaction (PCR) was performed using primer pair (Forward primer sequence: 5′ CATTACATGAAGTGGTAGGCAC 3′; Reverse primer sequence: 5′ CCCTCTTGTTAAGAACAACTGG 3′) covering exon 11 of *ERCC5* gene. PCR products were directly sequenced ABI Prism 3130 sequencer (Applied Biosystems, Foster City, CA, United States).

## Results

### Sanger Sequencing for Recurrent Mutations

Molecular investigation for recurrent mutations associated with XP mild phenotypes was performed using Sanger Sequencing. Indeed, as medical diagnosis firstly suggested an XP-A mild phenotype, the recurrent mutation p.Arg228^∗^ in *XPA* gene was searched for, with negative results. We then suspected that the disease was associated with other mild forms of XP with neurological manifestations. We thus searched for the *ERCC2* p.Arg683Gln mutation (XP-D phenotype) and the *DDB2* p.Lys381Argfs^∗^2 mutation (XP-E phenotype), which we previously identified in Tunisia. No variation was found in these patients. Therefore, samples underwent targeted gene high-throughput sequencing enlarged to all known DNA repair system genes.

### Targeted Gene Sequencing

**FIGURE 2:**
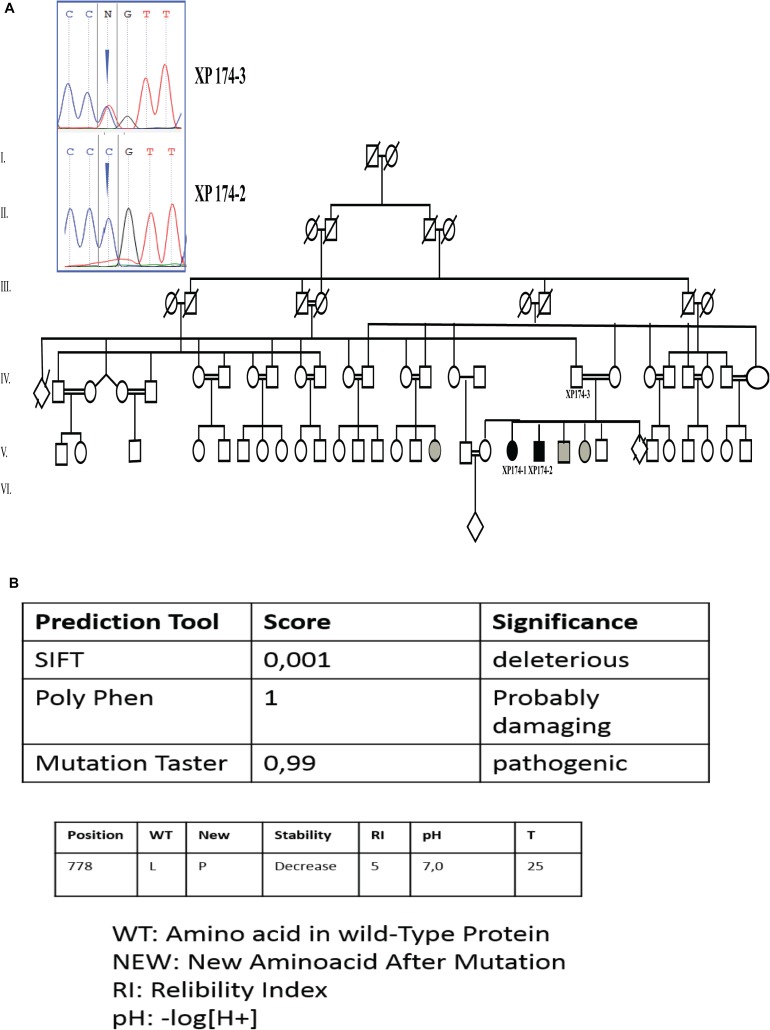
Genetic findings **(A)** pedigree describing affected family members and electropherogram showing the mutation in *ERCC5* gene, exon 11 (c.2333T>C p.L778P) at a homozygous state in the patients (XP174-1 end XP174-2) and at a heterozygous state in the father (XP174-3). Individual labeled in gray color are for suspected patients. **(B)** Prediction score through SIFT PolyPhen, mutation Taster, and I mutant.

Unexpectedly, we found a variation in the gene associated with XP-G form. Patient XP174-1 was a homozygous carrier of a missense variant in *ERCC5* gene (NM_000123.3) exon 11 (c.2333T>C; p.L778P). We thus performed Sanger sequencing on XP174-1 and XP17-2 to confirm our finding and genotype/phenotype correlation. We also checked for parental segregation in XP 174-3 (Figure 2).

Through online prediction tool of damaging effects of alternative amino acids changes like MutationTaster, SIFT, and POLYPhen, this variation was expected to be mysfunctional. I-Mutant v2.0 (Predictor of Protein Stability Changes upon Mutations), predicted decreased protein stability upon the p.L778P change (Figure 2).

It is interesting to note that we found a homozygous rs3818356 SNP in the intronic region of the *ERCC5* gene of these XP-G patients. Their father was heterozygous for this variant. Moreover, we found this variation in other patients from the same geographical region in whom a mild XP phenotype was suspected with no skin cancer development (data not shown), impelling further molecular explorations.

In order to assess the putative functional effect of rs3818356, we used the GTEx portal to evaluate the expression quantitative trait locus (eQTL) associations of this variant. Interestingly, significant eQTLs have been observed for rs3818356 that seems to alter the expression level of ERCC5 (*p* = 3.8 × 10-5) in skin tissue (Supplementary Figure S1).

## Discussion

Xeroderma pigmentosum is a rare genetic autosomal recessive disorder that affects the Tunisian population with a relatively high incidence compared to Europe (1:10.000 vs. 1:250000) (Zghal et al., 2005). More than 340 genetic diseases have been identified in the Tunisian population and the genetic etiologies are known for only 50% of these diseases (Romdhane et al., 2011).

Kasserine is a region in the Central West of Tunisia. Due to the high frequency of consanguineous unions, this region harbors many rare recessive genetic diseases (Romdhane et al., 2012). Regarding XP in this region, we observed a high frequency of XP-A patients carrying the p.R228^∗^ mutation presenting with a severe neurological phenotype, moreover a few families manifest mild forms of the disease like XP-D (Ben Rekaya et al., 2018). The emergence of an XP-G form that we identified here is also likely due to the high rate of consanguinity. Indeed, we have shown that consanguineous unions increase the risk of autosomal recessive diseases occurrence more than sixfold in the Tunisian population (Ben Halim et al., 2016).

It is also of note that patients who are affected by relatively mild sun sensitivity are usually under-diagnosed in Tunisia. Therefore, the use of high-throughput targeted gene sequencing to study diseases associated with deficient NER has proven to be extremely helpful to discover mutations that cause mild symptoms and result in very rare phenotypes (Calmels et al., 2016) like in the present case. This technology is a good alternative to whole exome sequencing patients whose clinical features are suggestive to involve DNA repair alterations.

Neurological symptoms associated with XP are mainly observed in complementation groups XP-A, XP-B, XP-D, and XP-G whereas XP-C, XP-E, and XP-V patients rarely exhibit neurological anomalies (Cleaver et al., 2009).

**FIGURE 3:**
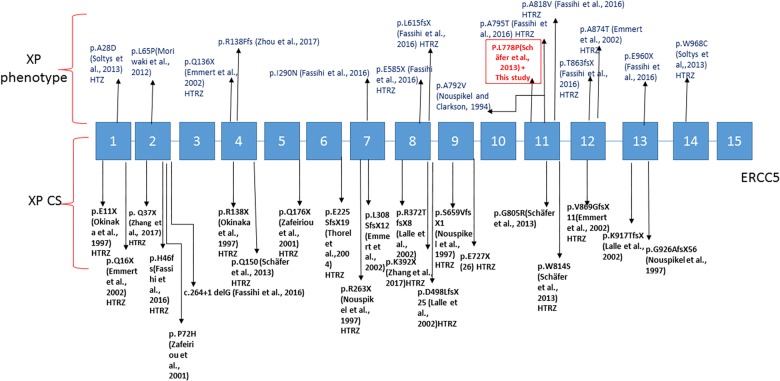
Linear map of the mutations in *ERCC5* gene associated with XP-G phenotype above and XP/CS below; variation with (HTZ) are for heterozygous patients. The boxed mutation is for the variation present in our case.

Based on complementation tests, XP-G is among the rarest groups of XP with only 42 patients reported to date worldwide. So far, only 36 genetic mutations have been identified (Norris et al., 1987; Emmert et al., 2002; Anttinen et al., 2008; Cleaver et al., 2009; Moriwaki et al., 2012; Schäfer et al., 2013; Soltys et al., 2013; Zhou et al., 2017; Supplementary Table S1 and Figure 3). The XP-G involves a wide range of clinical phenotype. Indeed, genetic mutations in the *ERCC5* gene are responsible for either a mild XP-G phenotype (reported in 21 patients) or a severe combined XP-CS phenotype (reported in 21 patients). No mutational hot spot has been reported to date in the *ERCC5* gene. In this work we found for the first time in Tunisia, two patients harboring a homozygous missense variant in exon 11 of the *ERCC5* gene (c.2333T>C; p.L778P). The father of the two siblings was carrier of this variant. Nevertheless, we were unable to reach the mother to confirm the homozygous state of the mutation in these patients. Hemizygosity or small deletion could not be excluded. However, several studies including ours (Romdhane et al., 2014) have shown that most individuals born from consanguineous marriages are homozygous not only for the deleterious variant but also for the whole segment inherited from a comment ancestor (Fareed and Afzal, 2017). Nevertheless, as the mother DNA was unavailable to check her status, we cannot rule out the hypothesis of a deletion inherited from her side.

The XPG nuclease is a structure-specific repair endonuclease, which cleaves DNA strands 3′ to the DNA damage site during nucleotide excision repair. It contains two domains (N and I) required for nuclease activity, separated by a large spacer domain. This variation is localized within the I domain of the nuclease that was suggested to interact with the XPD protein. However, the XPG protein interacts with XPD also via another region (1–377), and this second interaction may explain why the repair activity is to some extent maintained in patients carrying mutations in the I region (Iyer et al., 1996). The XPG-XPD interaction may also explain the similarities of the XP-G and XP-D mild phenotypes. Using prediction tools such as SIFT, Polyphen and Mutation taster, the variation NM_000123.3: c.2333T>C in the *ERCC5* gene was predicted to have a pathogenic effect. This missense variant has also been predicted to disrupt the helical structure of the protein in RAD2 homolog of XPG protein in a *Saccharomyces cerevisiae* model, which may affect RAD2 overall stability (Miêtus et al., 2014).

The variation identified in these two patients has been previously reported at the heterozygous state in fibroblasts of a patient cell line, XP40GO, for which neither clinical information nor the geographical origins were available. It was only reported that this patient carried two compound heterozygous variations in the *ERCC5* gene (exon 11 and exon 4) that could be responsible for the very severe XP/CS phenotype (Schäfer et al., 2013). The study conducted by Schäfer et al. (2013) with the XP40GO cell line assumed that the original patient had an XP-CS phenotype, since the authors found similar functional defects, using the Host Cell Reactivation (HCR) Assay in cells obtained from patients already diagnosed for the XP-CS disease.

However, using allele specific complementation analysis, Schäfer et al. showed that a residual repair activity was maintained for the (L778P) variation, while this was not the case for the other variants that were associated with XP-CS phenotype. We suggest the use of RRS (RNA recover synthesis) test that quantifies NER efficiency to predict the phenotype outcome between XP and combined XP-CS especially when clinical data are missing.

More interestingly, this case study is in accordance with the study conducted by Soltys et al. (2013), suggesting that patients harboring at least one allele that transcribes mRNA for non-truncated XPG have a mild XP-G clinical phenotype.

Patients with the XP-G phenotype reported in the literature generally develop skin cancers at a minimum age of 20 (Fassihi et al., 2016). However, these patients are generally protected from sunlight since childhood (as XP65BE, XP125LO patients) (Nouspikel and Clarkson, 1994; Emmert et al., 2002). The two siblings described here present a mild XP skin phenotype with no skin cancers, despite the lack of sun protection in a region where the UV index is high throughout most of the year. Moreover, these patients carried a second variation rs3818356 located in the intronic region of *ERCC5*. The high frequency of this SNP in the general population (MAF = 0.2738) strongly suggests that it corresponds to a non-deleterious variant. eQTL analysis showed that this variant increases significantly the expression level of ERCC5 in the skin. Alteration of gene expression levels may affect the associated phenotype, which may explain, at least in part, the mild XP-G phenotype observed in rs3818356 carriers. This rs3818356 has been associated with a better survival in hepatocellular carcinoma patients (Jung et al., 2012). In addition, we found rs3818356 in three other patients with similar moderate phenotype of XP (not yet classified); these patients did not develop skin cancers as well. The presence of this homozygous variant in our patients with mild XP phenotype suggests that it has a protective role against skin cancers. The association between p.L778P and the intronic variation rs3818356 in *ERCC*5 gene, combined with the interaction of XPG with other genetic modifiers in NER pathway might influence phenotypic outcome. Additional functional and association studies are needed to decipher these interactions.

Defects in the *ERCC5* gene remain a paradigm in DNA repair diseases. Indeed, mutations in this gene could result in phenotypes that range from mild XP form to the most severe combined form of XP/CS. It is noteworthy that, both XP and XP/CS are models for premature aging studies, especially for immune system senescence and skin aging investigations.

The impact of XPG deficiency in NER pathway, in sensitive cells like neurons and skin fibroblasts, may explain the neurological disorders and the premature aging. This hypothesis is being investigated in rodents (Vermeij et al., 2016) but this point is far from being elucidated, and further studies are also necessary in humans.

Thanks to the collaboration with the patients’ support group and the rise of awareness on XP and related diseases in North African populations, we noticed an increase of consultations for skin sensitivity. This situation could contribute to the identification of further milder forms of XP (XP-V, XP-D, XP-E, and XP-G) as in the cases we report.

In conclusion, the identification of a homozygous missense mutation causing XP-G adds a novel case to the panel of known XP forms in the Tunisian population and in North Africa. Our finding reveal that this genetic defect is one of the multiple causes that predispose to photo-aging, and that need to be deeply investigated in the future.

We propose to search for the newly reported XPG mutation in the molecular diagnostic workflow for patients originating from this geographical region, an upgrade for the identification tools to include targeted gene sequencing seems to be essential for better health support for XP disease in Tunisia.

## Author Contributions

AC did the experiments, analysis, and interpretation of data and drafted the manuscript. SE and AL did the experiments, analysis, and interpretation of data. MJ and MZ contributed to clinical investigation of patients and available family members. IN, MBR, and OM helped in patients pre-screening for known mutations. YH helped in *in silico* analyses. VD, ADS-G, NL, and SA contributed to study concept and design. ADS-G, SA, and HY-Y critically revised the manuscript. HY-Y supervised the study.

## Conflict of Interest Statement

The authors declare that the research was conducted in the absence of any commercial or financial relationships that could be construed as a potential conflict of interest.

## Footnotes

1 http://varaft.eu/index.php

2 http://evs.gs.washington.edu/EVS/

3 http://www.1000genomes.org/

4 http://exac.broadinstitute.org

5 http://www.mutationtaster.org/

6 http://genetics.bwh.harvard.edu/pph2/

7 http://sift.bii.a-star.edu.sg/

8 http://folding.biofold.org/i-mutant/i-mutant2.0.html

## Supplementary Material

FIGURE S1 eQtl analysis and boxplot representing the significant eQTL association between rs3818356 and ERCC5 in skin tissue. Red dot represent the significant *cis-*eQTLs (log10) for the queried gene (at FDR<5%). Gray dots are significant *cis-*eQTLs for all other SNP-gene pairs within the genomic region. The Box plots represent the expression levels of ERRC5 transcript with respect to the rs3818356 genotypes.

TABLE S1 List of reported ERCC5 mutations.

TABLE S2 List of the 87 gene used for targeted gene sequencing.
