# Supplementary figures and images for: Identification of a ERCC5 c.2333T>C (L778P) Variant in Two Tunisian Siblings With Mild Xeroderma Pigmentosum Phenotype
Source: Front Genet. 2019 Feb 14;10:111. doi: 10.3389/fgene.2019.00111 (PMC6383105; doi:10.3389/fgene.2019.00111)

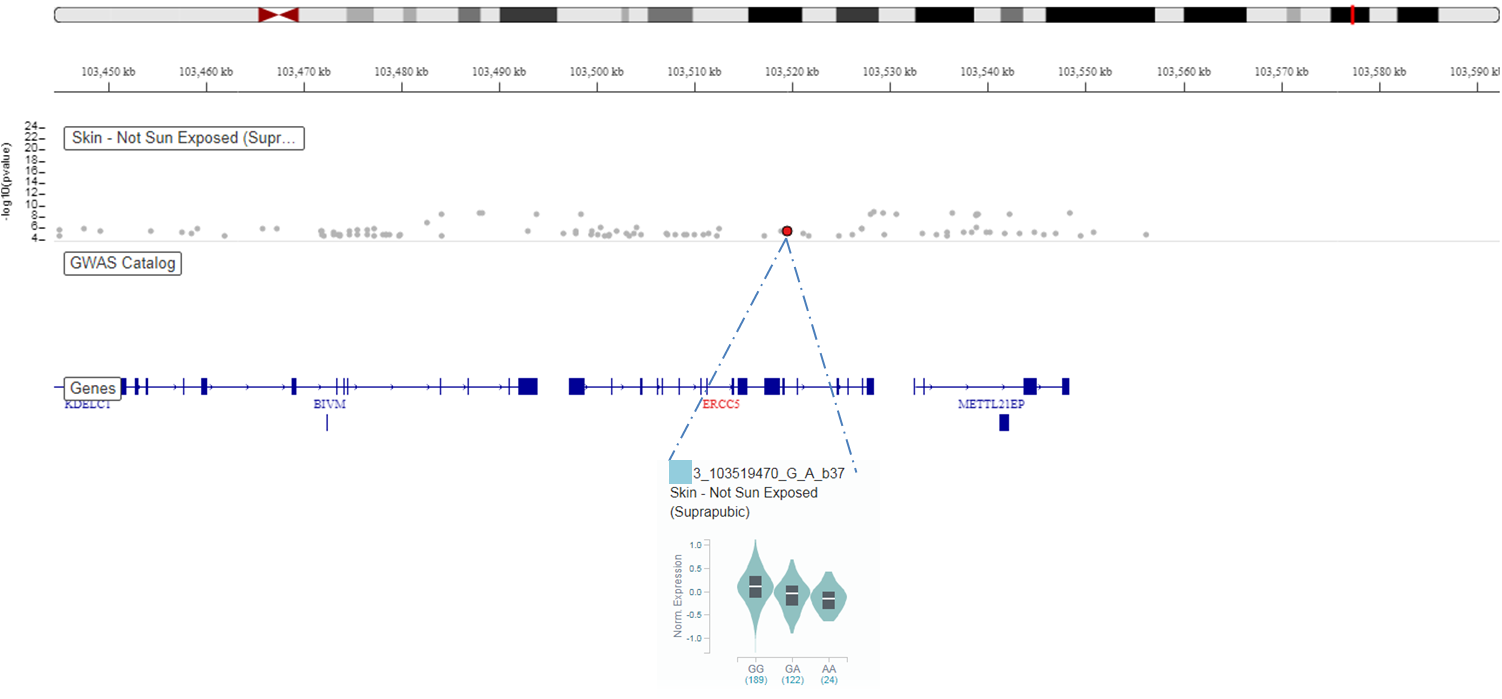

Supplement: FIGURE S1 — eQtl analysis and boxplot representing the significant eQTL association between rs3818356 and ERCC5 in skin tissue. Red dot represent the significant cis-eQTLs (log10) for the queried gene (at FDR<5%). Gray dots are significant cis-eQTLs for all other SNP-gene pairs within the genomic region. The Box plots represent the expression levels of ERRC5 transcript with respect to the rs3818356 genotypes. [file Image_1.TIFF]
