# Supplementary material for: Identification of a ERCC5 c.2333T>C (L778P) Variant in Two Tunisian Siblings With Mild Xeroderma Pigmentosum Phenotype
Source: Front Genet. 2019 Feb 14;10:111. doi: 10.3389/fgene.2019.00111 (PMC6383105; doi:10.3389/fgene.2019.00111)
Supplement: TABLE S1 — List of reported ERCC5 mutations. [file Table_1.docx]

**Supplementary material**

**Table 1:** List of reported ERCC5 mutations

| **Patients** | **Genotype and predicted translational products** | **Phenotype** | **Origin** | **Reference** |
| --- | --- | --- | --- | --- |
| XP125LO, XP124 LO | c.2375C>T (A792V) | XP-G | NA | (Nouspikel and Clarkson, 1994) |
| XPCS1LV | c.1977delA(S659VfsX1) | XP/CS | Flemish | (Nouspikel et al., 1997) |
| XPCS2LV (HTZ) | c.727C>T (R263X)  c.1977delA (S659VfsX1) | XP/CS | Flemish |  |
| 94RD27 | c.2779delT(G926Afs56) | XP/CS | Moroccan |  |
| XP20BE (HTZ) | c.30G>T (E11X)  c.412C>T(R138X) | XP/CS | NA | (Okinaka et al., 1997) |
| XP2BI (HTZ) | c.1115_1118delGGAA(R372Tfs)  c.2573T>C(L858P) | XP/CS | NA | (Lalle et al., 2002) |
| XP3BR (HTZ) | c.1494delA (K498LfsX25)  c.2751delA (K917NfsX65) | XP/CS | NA |  |
| XPCS4RO | c.215C>A(P72H)  c.526C>T (Q176X) | XP/CS | NA | (Zafeiriou et al., 2001) |
| XP3HM | c.194T>C(L65P) | Mild XP | Japanese | (Moriwaki et al., 2012) |
| XPCS1BD (HTZ) | c.(E225SfsX19) | XP/CS | NA | (Thorel et al., 2004) |
| XP96TA | c.921_922delTC(L308fsX12) | XP/CS | Israeli Palestinian | (Emmert et al., 2002) |
| XP82DC (HTZ) | c.46C>T(Q16X)  c.3049_3050delTG(V869GfsX11) | XP/CS | Caucasian |  |
| XP65BE (HTZ) | c.2620G>A(A874T)  c.406C>T(Q136X) | XP-G | Caucasian |  |
| XP12PF, XP13PF | c.409_410insTT(R138F) | XP-G | Chinese | (Zhou et al., 2017) |
| XP104BR | c.136delC (H46fs) | XP/CS | Pakistani | (Fassihi et al., 2016) |
| XP118BR (HTZ) | c.2383G>A(A795T)  c.1842delT(L615fs) | XP-G | Caucasian |  |
| XP55, XP56BR | c.264+1delG | XP/CS | Somalian |  |
| XP34BR (HTZ) | c.2453C>T(A818V)  c.2586_2587delTA(T863fs) | XP-G | Caucasian |  |
| XP120BR, XP119BR (HTZ) | c.2453C>T (A818V)  c.1753G>T(E585X) | XP-G | Celtic |  |
| XP101BR | c.869T>A(E960X) | XP-G | NA |  |
| XP01RJ, XP02RJ (HTZ) | c.83C>A (A28D)  c.2904G>C (W968C) | XP-G | Brazilian | (Soltys et al., 2013) |
| Infant girl (HTZ) | c.1173dupT (Q37X)  c.109C>T (L392X) | XP/CS | Chinese | (Zhang et al., 2017) |
| XP40GO (HTZ) | c.2333T>C (L778P)  c.448C>T (Q150X) | XP/CS [Predicted by Host Cell Reactivation (HCR) Assay] | NA | (Schäfer et al., 2013) |
| XP72MA (HTZ) | c.2440G>C (W814S)  c.2179G>T (E727X) | XP/CS | Caucasian |  |
| XP165MA | c.2413G>A (G805R) | XP/CS | Caucasian |  |

**Table 2:** List of the 87 Gene used for targeted gene sequencing

| **Disease group** | **Gene** |
| --- | --- |
| Albinism | C10ORF11 |
|  | GPR143 |
|  | MC1R |
|  | MITF |
|  | OCA2 |
|  | SLC45A2 |
|  | TYR |
|  | TYRP1 |
| DNA repair / chromosomal breakage | ATM |
|  | ATR |
|  | BLM |
|  | BRCA2 |
|  | DDB2 |
|  | ERCC1 |
|  | ERCC2 |
|  | ERCC3 |
|  | ERCC4 |
|  | ERCC5 |
|  | FANCA |
|  | FANCB |
|  | FANCC |
|  | FANCD2 |
|  | FANCE |
|  | FANCF |
|  | FANCG |
|  | FANCI |
|  | FANCL |
|  | FERMT1 |
|  | GTF2H3 |
|  | NBN |
|  | PALB2 |
|  | PCNA |
|  | POLH |
|  | RAD23B |
|  | RAD51C |
|  | RPA1 |
|  | SLX4 |
|  | SSRP1 |
|  | WRN |
|  | XPA |
|  | XPC |
|  | BRCA1 |
| Neurofibromatosis type1 and type 2 and Neurofibromatosis Like | MLH1 |
|  | MSH2 |
|  | NF1 |
|  | NF2 |
|  | PTPN11 |
|  | SDHB |
|  | SDHD |
|  | SPRED1 |
| Premature aging | ACD |
|  | BANF1 |
|  | CTC1 |
|  | DKC1 |
|  | ERCC6 |
|  | ERCC8 |
|  | LMNA |
|  | NHP2 |
|  | NLRP1 |
|  | NOP10 |
|  | PARN |
|  | RECQL4 |
|  | RTEL1 |
|  | TERC |
|  | TERT |
|  | TINF2 |
|  | USB1 |
|  | WRAP53 |
|  | ZMPSTE24 |
| Rasopathies | BRAF |
|  | CBL |
|  | HRAS |
|  | KRAS |
|  | LZTR1 |
|  | MAP2K2 |
|  | NRAS |
|  | RAF1 |
|  | RASA2 |
|  | RIT1 |
|  | RRAS |
|  | SHOC2 |
|  | SOS1 |
|  | SOS2 |
|  | SPRY1 |
|  | RASA1 |
| Tuberous sclerosis | TSC1 |
|  | TSC2 |

**Reference for Table 2:**

Emmert, S., Slor, H., Busch, D.B., Batko, S., Albert, R.B., Coleman, D., Khan, S.G., Abu-Libdeh, B., Digiovanna, J.J., and Cunningham, B.B. (2002). Relationship of neurologic degeneration to genotype in three xeroderma pigmentosum group G Patients1. *Journal of Investigative Dermatology* 118**,** 972-982.

Fassihi, H., Sethi, M., Fawcett, H., Wing, J., Chandler, N., Mohammed, S., Craythorne, E., Morley, A.M., Lim, R., and Turner, S. (2016). Deep phenotyping of 89 xeroderma pigmentosum patients reveals unexpected heterogeneity dependent on the precise molecular defect. *Proceedings of the National Academy of Sciences***,** 201519444.

Lalle, P., Nouspikel, T., Constantinou, A., Thorel, F., and Clarkson, S.G. (2002). The founding members of xeroderma pigmentosum group G produce XPG protein with severely impaired endonuclease activity. *Journal of Investigative Dermatology* 118**,** 344-351.

Moriwaki, S., Takigawa, M., Igarashi, N., Nagai, Y., Amano, H., Ishikawa, O., Khan, S.G., and Kraemer, K.H. (2012). Xeroderma pigmentosum complementation group G patient with a novel homozygous missense mutation and no neurological abnormalities. *Experimental dermatology* 21**,** 304-307.

Nouspikel, T., and Clarkson, S.G. (1994). Mutations that disable the DNA repair gene XPG in a xeroderma pigmentosum group G patient. *Human molecular genetics* 3**,** 963-967.

Nouspikel, T., Lalle, P., Leadon, S.A., Cooper, P.K., and Clarkson, S.G. (1997). A common mutational pattern in Cockayne syndrome patients from xeroderma pigmentosum group G: implications for a second XPG function. *Proceedings of the National Academy of Sciences* 94**,** 3116-3121.

Okinaka, R.T., Perez-Castro, A.V., Sena, A., Laubscher, K., Strniste, G.F., Park, M.S., Hernandez, R., Macinnes, M.A., and Kraemer, K.H. (1997). Heritable genetic alterations in a xeroderma pigmentosum group G/Cockayne syndrome pedigree. *Mutat Res* 385**,** 107-114.

Schäfer, A., Schubert, S., Gratchev, A., Seebode, C., Apel, A., Laspe, P., Hofmann, L., Ohlenbusch, A., Mori, T., and Kobayashi, N. (2013). Characterization of three XPG-defective patients identifies three missense mutations that impair repair and transcription. *Journal of Investigative Dermatology* 133**,** 1841-1849.

Soltys, D.T., Rocha, C.R., Lerner, L.K., De Souza, T.A., Munford, V., Cabral, F., Nardo, T., Stefanini, M., Sarasin, A., and Cabral‐Neto, J.B. (2013). Novel XPG (ERCC5) mutations affect DNA repair and cell survival after ultraviolet but not oxidative stress. *Human mutation* 34**,** 481-489.

Thorel, F., Constantinou, A., Dunand-Sauthier, I., Nouspikel, T., Lalle, P., Raams, A., Jaspers, N.G., Vermeulen, W., Shivji, M.K., Wood, R.D., and Clarkson, S.G. (2004). Definition of a short region of XPG necessary for TFIIH interaction and stable recruitment to sites of UV damage. *Mol Cell Biol* 24**,** 10670-10680.

Zafeiriou, D.I., Thorel, F., Andreou, A., Kleijer, W.J., Raams, A., Garritsen, V.H., Gombakis, N., Jaspers, N.G., and Clarkson, S.G. (2001). Xeroderma pigmentosum group G with severe neurological involvement and features of Cockayne syndrome in infancy. *Pediatric research* 49**,** 407.

Zhang, J., Cheng, R., Yu, X., Sun, Z., Li, M., and Yao, Z. (2017). Expansion of the genotypic and phenotypic spectrum of xeroderma pigmentosum in Chinese population. *Photodermatology, photoimmunology & photomedicine* 33**,** 58-63.

Zhou, E.Y., Wang, H., Lin, Z., Xu, G., Ma, Z., Zhao, J., Feng, C., Duo, L., Yin, J., and Yang, Y. (2017). Clinical and molecular epidemiological study of xeroderma pigmentosum in China: A case series of 19 patients. *The Journal of dermatology* 44**,** 71-75.
